# HPV Oncoproteins and the Ubiquitin Proteasome System: A Signature of Malignancy?

**DOI:** 10.3390/pathogens9020133

**Published:** 2020-02-18

**Authors:** Anamaria Đukić, Lucija Lulić, Miranda Thomas, Josipa Skelin, Nathaniel Edward Bennett Saidu, Magdalena Grce, Lawrence Banks, Vjekoslav Tomaić

**Affiliations:** 1 Division of Molecular Medicine, Ruđer Bošković Institute, Bijenička cesta 54, 10000 Zagreb, Croatia; Anamaria.Djukic@irb.hr (A.Đ.); Lucija.Lulic@irb.hr (L.L.); jskelin@irb.hr (J.S.); saidunebs@gmail.com (N.E.B.S.); grce@irb.hr (M.G.); 2 International Centre for Genetic Engineering and Biotechnology, AREA Science Park, Padriciano 99, I-34149 Trieste, Italy; miranda@icgeb.org (M.T.); banks@icgeb.org (L.B.)

**Keywords:** E6, E7, HPV, cervical cancer, proteasome, UPS, ubiquitin ligases, ubiquitin

## Abstract

Human papillomavirus (HPV) E6 and E7 oncoproteins are critical for development and maintenance of the malignant phenotype in HPV-induced cancers. These two viral oncoproteins interfere with a plethora of cellular pathways, including the regulation of cell cycle and the control of apoptosis, which are critical in maintaining normal cellular functions. E6 and E7 bind directly with certain components of the Ubiquitin Proteasome System (UPS), enabling them to manipulate a number of important cellular pathways. These activities are the means by which HPV establishes an environment supporting the normal viral life cycle, however in some instances they can also lead to the development of malignancy. In this review, we have discussed how E6 and E7 oncoproteins from alpha and beta HPV types interact with the components of the UPS, and how this interplay contributes to the development of cancer.

## 1. Introduction

*Papillomaviridae* is a diverse family of small, non-enveloped DNA viruses, approximately 50–60 nm in diameter that infect all homoeothermic vertebrates including humans [1,2]. Interestingly, recent studies have also detected members of *Papillomaviridae* in fish [3]. Currently, there are known to be approximately 200 different human papillomavirus (HPV) types, which are classified in five genera (alpha, beta, gamma, mu and nu) [4]. The *Alphapapillomavirus* species (α-HPVs) preferentially infect oral or anogenital mucosa in humans and primates [2]. The α-HPVs are further classified as low-risk (LR) (e.g., HPV-6 and HPV-11) or high-risk (HR) (e.g., HPV-16, HPV-18, and HPV-33), based on their association with human cancers [1]. HPV infections are usually fairly rapidly cleared by the immune system–from a few months up to two years from initial viral entry. However, in some instances, the infections are not neutralized by the immune system and they persist for long periods, which can result in the development of different types of neoplasia [5]. LR HPV types cause self-limiting benign anogenital warts and are only rarely found in squamous intraepithelial lesions, possibly as a part of multiple infections [6,7]. On the other hand, HR HPVs have been recognized as the main causative agent of cervical cancer, with more than 600,000 new cases annually worldwide [1,2,8,9]. HPV-16 and HPV-18 are associated with approximately 80% of cervical cancer globally, while the remaining 20% are linked to infection by other HR HPVs (e.g., HPV-31, HPV-33, HPV-45, and HPV-58) [1,8,10]. HPVs also cause different types of anogenital cancers and were shown to be associated with 30–50% of head-and-neck cancers (HNC), particularly affecting the oropharynx, the base of the tongue, and the tonsils [1,11,12,13,14,15]. Intriguingly, HPV-16 is the predominant cancer-causing type associated with the vast majority of HPV-positive HNCs, with other HR types being detected only sporadically [13,14].

The *Betapapillomavirus* genus comprises approximately 50 HPV types and can be detected in normal and precancerous tissue, as well as in cancerous cutaneous epithelial tissue [2,16]. Although β-HPVs are primarily considered to be non-carcinogenic in the general population, some β-HPV types, such as HPV-5 and HPV-8, can represent a potential cancer risk in particular hypersensitive groups (e.g., in cases of severe immunodeficiency and in epidermodysplasia verruciformis patients), where β-HPVs have been found in skin warts and in cutaneous squamous cell carcinomas (CSCC) [17,18]. In addition, these β-HPVs are also characterized as co-factors, which, in combination with ultraviolet (UV) irradiation, have been thought to contribute to the development of non-melanoma skin cancers (NMSC) [16]. This was later questioned and it was proposed that loss of T-cell immunity, rather than HPV activity, increased the risk of CSCC in immunosuppressed patients [19].

It is believed that HPVs infect cells in the basal layer of stratified squamous epithelia, exposed as a result of small abrasions and micro-wounds [20,21]. In normal epithelium, epithelial cells in the basal layer are proliferative, while the differentiating cells in the suprabasal layers have exited the cell cycle. The HPV life cycle is dependent upon the replicative machinery of basal cells, which have the potential to proliferate. Following infection, HPV genomes are established as extrachromosomal elements or episomes. Transition to the late stage of HPV infection is followed by the expression of late genes and virion production [20,22]. Although, in the majority of cases, HPV infection is asymptomatic or transient, in some individuals the viral genomes become integrated into the host DNA, with much of the viral genome being lost, which results in a breakdown of the productive life-cycle of the virus. In addition, integration of viral DNA usually leads to the upregulated expression of the early viral genes E6 and E7, resulting in increased proliferation of the infected cell, which is a crucial step in further malignant development [15,23,24]. However, there may potentially be integration that does not lead to increased E6/E7 expression, therefore not inducing a carcinogenic effect.

The β-HPV types infect the basal layer of the squamous epithelium and hair follicle region, but the mechanism remains unknown [25,26] although some β-HPVs, like HPV38, show similarities with mucosal HR types and were associated with cancer development through E7/Rb protein interactions [27,28]. Interestingly, while the viral integration of HR HPV types is considered to be one of the key events in the process of tumor formation and in maintenance of the transformed phenotype, this appears not to be the case with β-HPVs [29]. Studies have demonstrated that the process of viral integration is absent during β-HPV infections and there is no requirement for continual oncoprotein expression to allow the persistence of the transformed phenotype during CSCC, indicating that β-HPVs contribute to the initial steps of tumor formation, but are not necessary for its continued maintenance [16,17,28,30,31].

## 2. The Ubiquitin Proteasome System

The Ubiquitin Proteasome System (UPS) is the pathway responsible for the majority of intracellular protein degradation in eukaryotic cells. It controls the turnover of thousands of short-lived, regulatory, damaged and misfolded proteins, in order to regulate and maintain various cellular functions and cellular homeostasis [32]. It is a highly specific process and involves the addition of ubiquitin molecules to cellular substrates, which consequently leads to their modification via various cellular pathways. Fundamental cellular signaling pathways that are regulated by ubiquitination include cell cycle control, cell survival, cell proliferation, transcription, DNA repair, apoptosis, cellular metabolism, protein quality control, and membrane trafficking, as well as ubiquitination being very important in the immune response [33,34,35]. Ubiquitination, which involves a complex interplay of ubiquitinating and deubiquitinating enzymes (DUBs), is an essential element of control by the UPS [36,37]. Ubiquitin is transferred to target proteins by the ubiquitination cascade: first, ubiquitin is activated and bound by an E1 ubiquitin-activating enzyme, then transferred to an E2 ubiquitin-conjugating enzyme and, finally, transferred to lysine residues on the target protein by E3 ubiquitin-protein ligase [36,37]. So far, studies have identified two E1 activating enzymes, 40 E2 conjugating enzymes, around 800 E3 ubiquitin-protein ligases and about 100 DUBs [38]. While all components of the UPS are of great importance for ubiquitination, individual ubiquitin-protein ligases may be valuable viral targets as they are involved in substrate recognition, and hence define the specificity of the system. They are categorized into two main families: the numerous Really Interesting New Gene (RING) finger E3 ubiquitin ligases; and the less common Homologous to E6-associated protein C-Terminus (HECT) domain E3 ligases [39,40]. Polyubiquitin chains attached to substrates via lysine 48 will result in target protein degradation at the proteasome, while polyubiquitin chain attachment to other lysines will modulate proteasome-independent activities [41]. Proteins can also be mono-ubiquitinated, and this influences processes such as endocytosis and vesicular sorting [42]. On the other hand, ubiquitin molecules are cleaved from substrates by DUBs [43], which play essential roles in cellular processes as diverse as DNA replication, gene silencing, and endocytosis, ultimately affecting cell growth and oncogenesis. 

## 3. HPV and the UPS

Many human pathologies, such as inflammatory and neurodegenerative diseases and certain cancers are, directly or indirectly, promoted by the deregulation of the UPS [32,44]. An intriguing feature of both HPV oncoproteins, E6 and E7, is their ability to direct many of their cellular substrates for proteasome-mediated degradation [21,45]. Those interactions were either shown or confirmed using methods like co-immunoprecipitation, Glutathione-S-transferase (GST)-fusion protein pull-down, affinity chromatography, Yeast Two Hybrid Assay and/or Tandem Affinity Purification and *Gaussia princeps* luciferase protein complementation assay. HPV E6 and E7 proteins appear to have evolved various strategies to make use of the ubiquitin system to support the viral lifecycle [45,46,47]. In particular, to avoid apoptosis, E6 mainly targets p53 while, by targeting the pRb, p107 and p130 pocket proteins, E7 induces cell cycle progression [48,49,50]. E6 proteins from high-risk [51] and low-risk [52,53] HPVs are able to bind p53, and it was further shown that this binding promotes the degradation of p53 via the ubiquitin pathway [48]. Interestingly, E6 proteins from both high- and low-risk HPVs were also shown to have the capacity to bind p53 but without inducing its degradation [54,55]. Furthermore, a recent study showed that in the case of beta HPVs, HPV17a, HPV-38, and HPV-92 E6s could bind and/or stabilize p53 [56]. 

E6 and E7 oncoproteins inactivate the majority of these cellular substrates by interacting with components of the UPS, ultimately inducing their degradation at the proteasome. Among the diverse components of the UPS, the interplay between E6/E7 and ubiquitin ligases is one of the crucial aspects in this process. The best characterized ubiquitin ligases used by the two oncoproteins are E6-associated protein (E6AP) and the cullin-2 ubiquitin ligase complex [57]. E6AP complexes with E6 and is involved in targeting p53 and some other targets, while E7 uses the cullin-2 ubiquitin ligase complex to target pRb [48,58]. To date, this interaction has only been shown for HPV16 E7 [57]. There are also additional ubiquitin ligases and other components of the UPS, which are directed for similar activities by the viral oncoproteins [46,59,60,61,62,63]. Furthermore, HPV oncoprotein interactions with the UPS are not only critical for successful viral replication, but are also necessary for maintenance of the transformed phenotype [15,45]. Studies have shown that interference with components of the UPS has a down-regulatory effect on the ability of HPV-transformed cells to proliferate indefinitely and to avoid apoptosis [46,47,62,63,64]. Therefore, a better understanding of the interplay between HPVs and the UPS would provide important novel information and greatly improve our understanding of the viral life cycle and the process of HPV-induced tumorigenesis. 

## 4. HPV E6 Oncoprotein and the UPS

**Figure 1:**
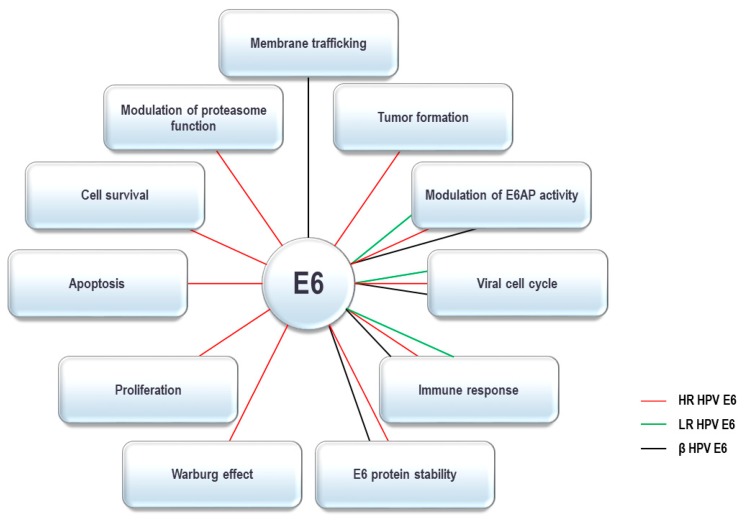
**The Ubiquitin Proteasome System (UPS) dependent activities of α- and β-HPV E6 oncoproteins**. E6 oncoproteins from α and β types interact with various components of the UPS and as speculated use them to modulate a number of cellular processes. By interacting with the UPS components high-risk (HR) α-type HPV E6s are involved in the regulation of all the processes shown above; low-risk (LR) α-type E6s are involved in regulation of viral life cycle, modulation of E6AP activity, and regulation of the immune response; β-type HPV E6s are involved in regulation of the viral life cycle, modulation of E6AP activity, regulation of the immune response, and E6 oncoprotein stability.

The HPV E6 oncoprotein contains around 150 amino acids and has two zinc fingers created by four CXXC motifs [65,66,67]. The integrity of these motifs is crucial for optimal oncoprotein function and they are highly conserved between all HPV E6 oncoproteins identified so far [68,69]. Recent studies have characterized the crystal structure of the intact E6 oncoprotein and also support the fact that E6 forms interactions with a large number of cellular substrates [67,70,71,72,73]. Recent studies have characterized the crystal structure of the intact HPV-16 E6 oncoprotein and specific domains of HPV-18 E6, HPV-51 E6 and Bovine Papillomavirus 1 (BPV-1) E6, and these data all show that E6 forms interactions with a large number of cellular substrates [70,72,74]. HPV E6 complexes with these substrates through a number of conserved binding motifs. One of these is the so-called PDZ-binding motif (PBM), exclusively present on the C-terminus of HR HPV E6 oncoproteins, through which they bind to numerous PDZ-domain containing proteins [10,61]. Another conserved binding motif on E6 is the LXXLL binding motif. The most notable E6 targets with an LXXLL motif include E6AP, the preferred interacting partner of α-HPV E6 oncoproteins, and MAML-1, interacting with β-HPV E6 oncoproteins [75,76,77,78,79,80]. As mentioned above, E6 oncoproteins from both α- and β-HPV types interact with multiple components of the UPS; these interacting partners and their corresponding cellular functions are summarized in Table 1. By interacting with UPS components, HPV E6 oncoproteins can modulate various cellular functions, which are shown in Figure 1 and will be discussed in more detail below.

### 4.1. E6 Oncoprotein and Ubiquitin Ligases

HPV E6s interact with a number of cellular ubiquitin ligases. E6AP or UBE3A E3 ubiquitin-protein ligase is the principal ubiquitin ligase that associates with α-HPV E6 proteins. The interaction occurs via an LXXLL binding motif on E6 and leads to stimulation of E6AP ubiquitin ligase activity [81,82,83]. This association forms a stable complex between the viral oncoprotein and the ubiquitin ligase, which then targets a number of cellular substrates for proteasome-mediated degradation, with the p53 tumor suppressor being the most important cellular target [84]. This activity results in modulations of various cellular pathways to optimize the cellular environment for a productive viral life cycle, and in rare cases can initiate the process of carcinogenesis (Figure 1) [75,84,85,86]. Furthermore, a series of studies have also indicated that the general transcriptional effects of E6 are mostly dependent on the presence of E6AP [87]. This observation was further analyzed and supported by more recent studies, which demonstrated that the stability of α-HPV E6 oncoproteins was strictly dependent on the presence of E6AP [47]. Since E6AP was initially identified through its interaction with HR HPV E6-16 and HPV-18 proteins, it was originally thought that this association was exclusive to HR HPV mucosotropic types [84,85], but further studies showed that a LR HPV type 11 E6 could also complex with E6AP [83,88]. In addition, in vitro proteomic analysis and mass spectroscopy of cutaneous HPV cellular binding partners has shown that E6AP is a pulldown partner of E6 proteins from cutaneous-specific HPV-10 (an α-HPV) and HPV-24 (a β-HPV), indicating that β-HPV types can also interact with E6AP [82]. Furthermore, β-HPV types 24 and 38 E6 were also shown to form a complex with E6AP [77,81,82,88]. Interestingly, the interaction of E6AP with HPV-10 and HPV-24 E6 oncoproteins also increases their cellular protein levels indicating a more general dependence of all HPV types on E6AP for the maintenance of appropriate levels of E6 within the cell [82].

HPV-16 E6 oncoprotein was also shown to interact with HERC2, another putative HECT domain-containing E3 ubiquitin ligase [46,89]. Interestingly, it was found that E6AP physically mediates HPV-16 E6 and HERC2 interaction, which, in turn, can modulate the ubiquitin ligase activity of E6AP [90]. Subsequently, it was also shown that HERC2 can bind to E6AP in the absence of E6 [91]. Follow-up studies have demonstrated that, besides HPV-16 E6, several other HPV E6 oncoproteins (from HPV types 33, 52, 18, 45, 6b and 17a), including HR and LR HPV types, as well E6 oncoproteins from certain cutaneous β-HPV types, also interact with HERC2 [77]. However, the role of HERC2 in the HPV life cycle, and its contribution to the process of HPV-induced malignancy have yet to be elucidated [90].

UBR5/EDD is another HECT domain-containing E3 ubiquitin ligase, alterations in which have been linked to carcinogenesis [92,93]. Studies have shown EDD to be bound strongly by HPV-18 E6, but only weakly by HPV-16 and HPV-11 E6, suggesting that the interaction between E6 and EDD may be restricted to HPV-18 E6. EDD protein alone can bind independently to both E6 and E6AP, and it is also involved in regulating the E6/E6AP complex [94]. It appears that EDD regulation of E6AP expression is independent of E6, but that loss of EDD stimulates the proteolytic activity of the E6/E6AP complex, significantly increasing the ability of the E6/E6-AP complex to direct the degradation of its cellular substrates, particularly p53. Thus fluctuations of EDD protein levels might directly affect the viral life cycle, and influence the development of HPV-induced malignancies [94]. In addition, it has also been shown that the association of EDD with HR HPV E6 is important in destabilizing TIP60; a histone acetyltransferase tumor suppressor, thus ultimately contributing to the development of HPV-induced malignancies [95].

Another E3 ubiquitin ligase that has been shown to interact with HPV E6 oncoproteins is TRIM25, which is involved in immune system regulation [96,97]. Different HPVs E6 were shown to interact with TRIM25, including HR HPVs (HPV-16, -18, -33, and -52 E6), LR HPVs (HPV-6 and -11 E6) and cutaneous β-HPVs (HPV-5 and -8 E6), suggesting that HPV involvement in modulation of immune surveillance is conserved between different HPV types [98]. 

The BARD1 protein is a RING heterodimer that interacts with BRCA1, providing the E3 ubiquitin ligase activity that is required for BRCA1’s tumor suppressor function, as well as coordination of ubiquitination to maintain genomic stability [99,100]. BARD1 is a binding partner of HR HPV-16 and -18 E6, and it only interacts with these two HR HPV types, while no association was observed with LR HPV-11 E6 [101]. Furthermore, HR E6 oncoproteins were shown to interact directly with BRCA1 in both in vitro and in vivo conditions [102]. Interestingly, these interactions do not stimulate any degradation of either BRCA1 or BARD1, but the E6-induced increase in hTERT activity was considerably greater in the presence of BRCA1 than in its absence, suggesting that E6 possibly functions in part to antagonize the activity of BRCA1 [102]. However, it still remains to be clarified whether BRCA1 plays a role in the initiation of cervical cancer. In a recent study, Poirson et al. [38] using the *Gaussia princeps* luciferase protein complementation assay (GPCA), identified a number of cellular ubiquitin ligases or substrates with ubiquitin ligase activity as interacting partners of HPV E6 [38]. These studies assembled and screened a library of 590 cDNAs related to the UPS that covered about 50% of the human ubiquitination system, together with co-immunoprecipitation to confirm novel cell target protein-E6 interaction. Results indicated various new target proteins, including three RING-type Ub ligases MGRN1, LNX3 and LNX4 [38,103]. MGRN1 was shown to interact with E6 proteins from LR HPV-6 and HR HPV types 16, 18 and 33, as well as β-types HPV types 8 and 38. Interaction with LNX4 was restricted to HPV-16 E6, while LNX3 interacted with E6 oncoproteins from HR HPV types 16, 18 and 33 and β-HPV type 8 E6. The other ubiquitin ligases examined in this study were shown to exclusively bind HPV-16 E6, and they include ITCH, TRAF6, TRAF5, UBAC1, VHL, XIAP, RNF25 and RNF40 [38]. These ubiquitin ligases are associated with the regulation of several different cellular pathways, however, biochemical and mechanistic analyses to elucidate their roles in the viral life cycle and in HPV-mediated malignancies are still lacking [38,104]. 

Interestingly, there are also ubiquitin ligases that interact exclusively with β-cutaneous E6 oncoproteins, for example, HPV-17a and HPV-3 E6 which were reported to interact with 10 subunits (CNOT1, CNOT2, CNOT3, CNOT4, CNOT6L, CNOT7, CNOT9/RQCD1, CNOT10, C2orf29, and TNKS1BP1) of the Ccr4-Not multiprotein complex, which has been shown to be associated with various enzymatic activities including a ubiquitin ligase function [77,105]. White et al. detected those interactions by using an unbiased proteomic study including immunoprecipitation and mass spectrometry [77]. In addition, an E3 ubiquitin-protein ligase, UBR4/p600 was characterized as an interacting partner of cutaneous β-HPV-38 E6 [82], which has been shown to promote tumorigenesis in transgenic mouse models [28,106]. Moreover, this association was also observed but not validated in the proteomic analysis of another study [77]. Interaction between E6 and p600 was quite surprising, since p600 had been previously reported only as interacting partner of E7 [62,63], and more recent studies have shown that high risk E7 oncoproteins use p600 to target the PTPN14 tumor suppressor for proteasome-mediated degradation [59,107,108]. Therefore, it would be of a great interest to investigate the possible role of p600 in HPV-38-induced malignancy. Finally, a study by Holloway et al. [60] showed that β-HPV-5 E6 uses a specific and unique mechanism to target the proapoptotic factor BAK by recruiting the HECT domain-containing E3 ubiquitin ligase HERC1, which binds the apoptosis-promoting BAK protein in UV-damaged, E6-expressing cells. HERC1 specifically targets BAK in its active conformation, and only in the presence of E6, suggesting that HPV-5 E6 interacts with HERC1 to redirect its activity towards activated BAK, specifically to abrogate apoptosis of the infected cell and maintain the virus lifecycle [60]. 

### 4.2. E6 Oncoprotein and Deubiquitinating Enzymes

Apart from ubiquitin ligases, there are also other elements of the UPS, such as DUBs, that have been shown to interact with E6 oncoproteins from α- and β-HPV types. One of these is CYLD lysine 63 (K63) deubiquitinase, a negative regulator of the NF-κB pathway, which was shown to form complexes with HR HPV-16 and -18 E6, although there was no evidence for direct interaction [109]. Nonetheless, a study demonstrated that CYLD polyubiquitination and degradation under hypoxic conditions was E6-mediated. This implies that this mechanism, which E6 employs under hypoxic conditions, is likely to contribute to an aggressive microenvironment in tumors caused by HPVs; it also suggests that prolonged hypoxia-induced NF-κB activation may be specific only to HPV-induced cancers, such as cervical and head-and-neck cancers [109].

HPV E6 oncoproteins were further reported to interact with two other DUBs: ubiquitin-specific protease (USP) enzymes, USP15 and USP46 [38]. USP15 was shown to be bound by E6 proteins from α-HPV types 16, 18, 33 and 6, and β-HPV types 8 and 38. Biochemically it was demonstrated that USP15 was involved in the regulation of HPV-16 E6 protein stability [98]. Furthermore, HPV E6 protein forms a ternary complex with USP15 and TRIM25, resulting in the inhibition of immune surveillance and antiviral responses, thereby exhibiting a direct involvement in immune system regulation [98]. Recently, it was demonstrated that HR HPV-16, -18 and -31 E6 bind to USP46, but that no interactions were seen with the LR HPV types [110]. Another study also showed that USP46 is required for the proliferation of HPV-transformed cancers and derived cancer cells, indicating that USP46 can be essential for the survival of HPV-transformed cancers [110]. 

### 4.3. E6 Oncoprotein and the Proteasome 

HPV E6 oncoproteins from α- and β-types were also shown to bind directly to the proteasome. In vitro studies using GST-pull down assays have demonstrated that HR HPV-18 E6 interacts with several proteasomal subunits, including PSMC1, PSMC5, PSMD2, PSMC3, PSMC4, and PSMC2, while under the same conditions HR HPV-16 and LR HPV-11 E6 interacts with both subunits PSMD2 and PSMC1 [111]. Intriguingly, these interactions seem to be independent of E6AP, but the presence of E6AP does appear to be required for E6 to interact with PSMD4. This tripartite complex enhances the ubiquitination of PSMD4, an essential subunit of the 19S proteosome regulatory complex [111]. The HPV E6 oncoproteins from many HPV types can interact with diverse proteasome subunits, albeit they differ in their interaction profiles and their preferences for binding different proteasome subunits. These observations were supported by a series of analyses that demonstrated interactions between multiple α-HPV E6 oncoproteins and PSMA3, PSMC2, PSMC3, PSMD1, PSMD2, PSMD3, PSMD14, PSMD11, PSMD7, PSMC4, PSMD13, PSMD6, PSMD8, PSMB7, PSMB9 and PSME4 proteasomal subunits, while a panel of β-HPV E6 oncoproteins interacted only with a few proteasomal subunits, including PSMA3, PSMC2, PSMD1, PSMD2, PSMD3, PSMD11, and PSMD13 [77,112,113]. Those analyses included different methods, including affinity chromatography [113], immunoprecipitation and mass spectrometry [77], as well as the yeast two-hybrid (Y2H) system and tandem affinity purification [112]. Hence, E6 proteins from α- or β-HPVs exhibit different affinities of binding to various proteasomal subunits [77]. These observations were additionally confirmed by several other studies [90,113,114,115,116,117], suggesting the importance of the close association of E6 with the proteasome, probably through E6AP which directly connects E6 to the proteasome [77]. 

## 5. HPV E7 Oncoprotein and the UPS

HPV E7 oncoprotein is a small acidic protein of approximately 100 amino acid residues, having no significant sequence similarities to any cellular protein, except for the LXCXE motif. The amino terminus contains two regions similar to the E1A adenovirus proteins: the conserved region 2 (CR2) and part of CR1 [65]. The E7 oncoprotein also contains CR3, a zinc-binding site formed by two CXXC domains that function as a dimerization domain [118]. The CR3 domains are similar to those of the HPV E6 protein, suggesting that a genetic duplication may have occurred early in HPV evolution [119]. E7 has, in addition, sequences related to those of simian vacuolating virus 40 large tumor antigen (SV40 T), all contributing to the transforming activities of HR HPV E7 oncoproteins [120,121,122,123]. 

**Figure 2:**
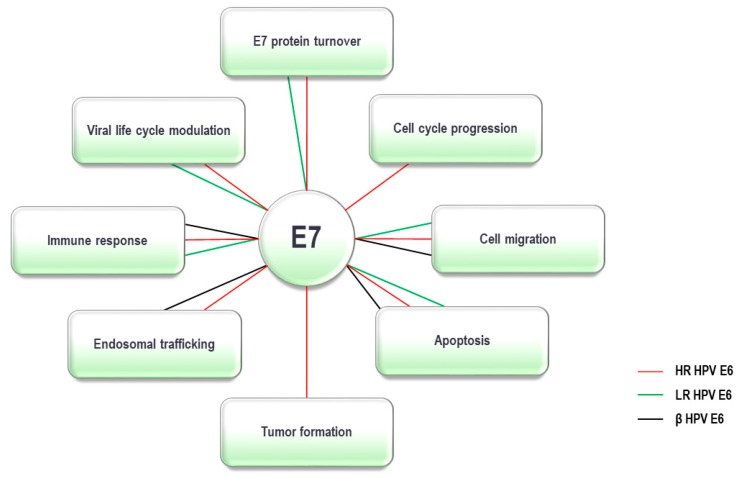
**The Ubiquitin Proteasome System (UPS) dependent activities of α- and β-HPV E7 oncoproteins**. E7 oncoproteins from α and β types interact with various components of the UPS and are thought to use them to modulate a number of cellular processes. By interacting with the UPS components, high-risk (HR) α-type E7s are involved in the regulation of all the processes shown above; low-risk (LR) α-type E7s are involved in cell migration, apoptosis, regulation of the immune response, viral life cycle modulation and E7 protein turnover; β-type E7s are involved in cell migration, apoptosis, endosomal trafficking and regulation of the immune response.

The main function of the HPV E7 oncoprotein is to maintain the infected differentiating cell in a DNA replication-competent state, which it does in part, by targeting the retinoblastoma tumor suppressor (pRb), a critical regulator of cell cycle progression, which controls the G1 to S-phase transition [124]. E7 also binds to the pRb-related members of the pocket protein family, p107 and p130, which assists in driving the cell cycle of the differentiating HPV-infected epithelial cell into an S-phase-like state, an environment suitable for replication of the viral genome [125]. In order to complete these activities, E7 forms interactions with ubiquitin ligases, which are essential elements of the UPS. In addition, E7 was shown to complex with other components of the UPS, and these interactions appeared to be important either for the stability of the oncoprotein or for its role in regulation and modulation of many cellular processes, some of which are summarized in Table 2 and Figure 2.

### 5.1. E7 Oncoprotein and Ubiquitin Ligases

A large number of ubiquitin ligases have been reported to be HPV E7 interactors, a principal one being cullin 2 (CUL2), a core component of the cullin-RING-based ESC (ElonginBC–Cullin–SOCS-box) E3 ubiquitin-protein ligase complex [126,127]. This interaction occurs via the E7 CR1 domain, as well as through its C-terminal sequences, and drives cell cycle progression by degradation of pRb and upregulation of CDK2 and cyclins A and E [58,128]. In fact, interactions between E7 and CUL2 complex were validated for 17 different HPV types from both the α- (HPV-16, -18, -31, -33, -45, -6b, -55, -74, -2a, and -57) and β-genus (HPV-8, -25, -98, -17a, -38, -76, and -92) HPV types. Furthermore, it has been shown that HPV-16 E7-expressing cells require ZER1 (or Zyg11BL), a substrate-specificity factor for a CUL2-RING ubiquitin ligase [57,129]. ZER1 is required for HPV-16 E7 to bind CUL2 and destabilize pRb, suggesting that HPV-16 E7 exploits the CUL2–ZER1 complex for pRb degradation. Interestingly, the ZER1 association appears to be specific only to HPV-16 E7. This may be related to the fact that ZER1 contains BC and a CUL2-binding site and acts in a complex with elongin B, elongin C, and CUL2, while HPV-16 E7 interacts with CUL2, and elongin C is required specifically for the binding of ZER1 to CUL2 [58,130]. Thus, all three proteins interconnect through a number of possible protein interactions.

E7 oncoprotein, similarly to E6, generally has a short half-life, approximately one hour [124]. It has been shown that E7 is ubiquitinated by the UBE2L3/Cullin 1 (CUL1) complex, followed by degradation at the proteasome and, to date, this has been observed only with HPV-16 E7 (122–124). CUL1 is a core component of multiple cullin-RING-based SKP1-CUL1-F-box proteins (SCF) E3 ubiquitin-protein ligase complexes, which were previously described not only to degrade E7 itself, but also to cause destabilization and degradation of the p130 pocket protein through its interactions with E7 [131,132,133,134,135]. Therefore, E7 interacts with the SCF ubiquitin ligase complex containing CUL1 and Skp2 and can be ubiquitinated by the CUL1-containing ubiquitin ligase in vitro and in vivo [131]. Finally, the half-life of E7 was found to be significantly longer in Skp2 (-/-) mouse embryo fibroblasts (MEFs) than in wild-type MEFs. Interactions were also observed between cullin 3 (CUL3) and E7 from HPV types 16, 18, 6b, 55 and 8, with a suggestion that the E7 and CUL3 may not interact directly, but probably via some BTB proteins [57]. In addition, E7 protein turnover is shown to be driven by ubiquitin-dependent proteolysis and the process itself is regulated by the two E2 ubiquitin-conjugating enzymes, UbcH7 and UBE2A [38,131]. While there are no solid reports about UbcH7 functions, it is known that UBE2A plays roles in a number of different biological processes and its binding partners include KCMF1 and UBR4/p600 [136]. Their roles in HPV E7 modulations of various cellular functions will be discussed in more detail further in the review.

The HPV-16 E7 protein binds to eleven POZ/BTB (Pox virus and Zinc finger/Bric-a-brac Tramtrack Broad complex) domain-containing proteins (BTBD15, KCTD13, NAC-1/NACC-1, TNFAIP1, SHKBP1, ZBTB9, ZBTB20, ZBTB32, ZBTB42, ZBTB43 and ZBTB48) [38]. The functions of these BTB proteins are largely unknown, and a number of different functional roles have been reported for the POZ/BTB domain, including transcriptional repression [137,138], cytoskeleton regulation [139,140], and protein ubiquitination [141,142,143]. POZ/BTB proteins interact with the CUL3-SCF-like E3 ubiquitin ligase complex, allowing the POZ/BTB domain to recruit substrate molecules for ubiquitination by the CUL3 component of the SCF-like complex [126,144,145]. Furthermore, some BTB domain-containing proteins have been identified as substrate adaptors of certain CUL3-RING Ub ligases, such as tumor necrosis factor α-induced protein 1 (TNFAIP1) and the homologous KCTD13 [146]. In addition, some BTB proteins also have a C2H2-type zinc finger (ZBTB9, ZBTB32, BTBD15, ZBTB48), which is frequently found in transcriptional activators, and this is consistent with E7 being a major transcriptional regulator [147]. The majority of these UPS proteins have only been characterized as E7 interacting partners, and importantly, the biological consequences of these interactions have yet to be investigated (Table 2). For example, NAC-1 is a POZ/BTB domain-containing transcriptional repressor protein related to tumor recurrence, and is essential for tumor growth and survival. The interaction between the BTB/POZ domains of NAC-1 is critical for tumor cell proliferation [143,148]. NAC-1 has been reported to be highly expressed in a number of human carcinomas, and, especially interestingly, NAC-1 is overexpressed in oral squamous cell carcinoma cells from different oral lesions [149,150]. Furthermore, HPV infection induces cytokine production, with TNF-α and related cytokines inducing various signaling pathways leading to growth arrest, proliferation, or cell death, but can also cause the expression of TNFAIP1, another POZ/BTB domain-containing protein. HPV-16 E7 oncoprotein was found to bind TNFAIP1, leading to TNF-α-mediated apoptosis [151].

Among the binding partners that interact with the E7 CR1 is p600 (ubiquitin N-recognin domain-containing E3 ligase 4) [57,62], a unique 600-kDa retinoblastoma-associated factor that regulates caspase-mediated programmed cell death known as anoikis [63,107]. The ability of E7 to associate with p600 is independent of its binding to the pocket proteins and occurs via the N-terminal domain of E7, thus contributing independently to cellular transformation [62]. Reduction of p600 protein levels results in a marked decrease in anchorage-independent growth both in HPV-positive and HPV-negative cancer cells, suggesting that p600 is normally involved in regulating anchorage-independent growth and cellular transformation. Furthermore, p600 is also involved in the HR E7-induced proteasome-mediated degradation of PTPN14 in cells derived from cervical tumors [57,59], and this process is independent of cullin-1 or cullin-2 [57,59]. In support of this, later studies have suggested the requirement for p600 in various processes involving viral life cycle modulation and the development of malignancy, through its interaction with HPV-16 and HPV-18 E7 [57,62]. In addition, p600 has been shown to bind various α- (HPV-16, -18, -31, -33, -45, -6b, -55, -74, -2a, and -57) and β-genus (HPV-8, -25, -17a, -38, and -92) HPV E7 oncoproteins [57]. Since it is clear that p600 binds to E7 proteins from numerous HPV E7 types, including benign HPV types, this interaction may also have major importance for the virus life cycle. Furthermore, p600 can interact with KCMF1 in the absence of E7, suggesting the possibility that E7, KCMF1, and p600 form part of a larger complex in cells. KCMF1 is a RING E3 ubiquitin-protein ligase and promotes ubiquitination [136]. Furthermore, the interaction between E7 and KCMF1 is also highly conserved across different HPV types, including interactions with various α- (HPV-16, -18, -31, -33, -45, -6b, -55, -74, -2a, and -57) and β-genus (HPV-8, -25, -17a, -38, and -92) HPV E7 oncoproteins [57].

Moreover, HPV E7 was found to interact with TNF receptor-associated factors (TRAFs), a family of E3 ubiquitin ligases associating with the TNF receptor superfamily, and contributing to the activation of NF-κB and MAP kinases [152]. TRAF2, an anti-apoptotic protein and an essential constituent of several E3 ubiquitin-protein ligases complexes, was shown to interact with HR HPV types 16, 18 and 33 E7 oncoproteins and also with β-HPV types 8 and 38, while no interaction was detected for LR HPV E7 oncoproteins [38]. A later study determined that HPV-16-infected keratinocytes exhibited an increase in TRAF2 expression and, more specifically, TNF receptor profile changes from type 1 to type 2, as well as the cells being more resistant to TNF-α induction of apoptosis [153]. This is probably responsible, at least in part, for the switch from an apoptotic to a proliferative fate, seen in HPV-16 infected keratinocytes [153]. Conversely, a unique adaptor protein and ubiquitin ligase, TRAF3, was found to interact only with HR HPV types 16 and 18 E7 oncoproteins [38]. A further study showed that increased expression of TRAF3 enhances p53 and pRb expression and decreases HPV E6 expression in HPV-positive cells, thus inhibiting cell growth, colony formation, migration, and enhances susceptibility to TNF-α and cisplatin-induced cell death [154]. These findings suggest that TRAF3 might have a tumor suppressor role, modulating established cancer hallmarks in HPV-infected cells [154]. Although interactions of TRAF4 and TRAF5 with E7 were detected, their biological effects on the host cell are yet to be studied [38]. TRAF5 is also an interacting partner of HR E6, as well as interacting with the E7 of all HPV types examined, including HPV-16, -18, -33, -6, -8 and -38.

Some other ubiquitin ligases that are involved in pathogen recognition and which associate with E7 are members of the TRIM family [38]. This comprises a number of proteins involved in many biological and antiviral processes, and E7 oncoproteins from many different HPV types interact with TRIM9, TRIM22, TRIM32 and TRIM72; some of these interactions have been further characterized and confirmed by co-immunoprecipitation, including that between E7 and TRIM32 [38]. TRIM32 is a RING finger Ub ligase implicated in innate immunity and has potential to bind E7 from HR α-HPV-16, -18 and -33, LR HPV-6, and also β-HPV types 8 and 38. TRIM72 has demonstrated the potential to bind E7 oncoproteins from certain HR α-HPVs (HPV-16, -18, and -33) and β-HPVs (HPV-8, and -38), but not a LR HPV-6 E7 oncoprotein [38]. Only TRIM22 has been fully characterized and seems to be downregulated in HPV-16- and HPV-18-positive cervical cancers [155].

Additional interactions have been identified between the E7 oncoproteins of LR HPV-6, HR HPV-16, -18, -33, and β-HPV-8 and -38 and RING ubiquitin ligase NEURL1, while only HR (HPV-16, -18 and -33) and LR (HPV-6) E7 binds to another RING ubiquitin ligase RNF135 [38]. However, the consequences of these interactions have yet to be investigated. Furthermore, interactions between E7 oncoproteins and ubiquitin ligase SH3RF1 were detected via GPCA assay, but have not been additionally confirmed by other interaction assays (34). 

### 5.2. E7 Oncoprotein and Deubiquitinating Enzymes

E7 oncoprotein also interacts with some DUBs; amongst these are USP26 and USP33, which are ubiquitin carboxyl-terminal hydrolases known to be involved in the ubiquitin-dependent proteolytic degradation of proteins via the 26S proteasome. Both USP26 and USP33 were identified as interactors of HR HPVs (HPV-16, -18 and -33), but not of β-HPVs (HPV-8 and -38), and have not so far been investigated in detail [38]. The USP29, a DUB implicated in thiol-dependent hydrolysis, also interacts with HR HPV-16, -18 and -33 E7 oncoproteins [38].

USP11, which belongs to a class of DUB that cleaves polyubiquitin chains, and thus inhibits the proteasome-mediated degradation of target proteins, is on the other hand, the only USP component so far shown to be bound by E7. USP11 increases the steady state levels of HPV-16 E7 by attenuating E7 ubiquitination and degradation, thus protecting E7 as well as influencing its effects on cell proliferation [156]. This interaction results in an increased stability and prolonged half-life of E7, but also suggests its requirement for modulation of downstream target proteins, subsequently affecting the biological function of E7, as well as abrogating its contribution to cell transformation. 

### 5.3. E7 Oncoprotein and Proteasome Components

Recent studies have shown that the levels of E7 protein in cancer cells are regulated by ubiquitin-dependent proteolysis via the 26S proteasome [132,135,157]. Furthermore, E7 seems to interact with the 26S proteasome subunit 4 (S4) ATPase via E7’s carboxyl-terminal zinc binding motif; the interaction thus being independent of the E7-pRb interaction. Therefore, E7 might target pRb for degradation by directly interacting with the 26S proteasome through S4. Moreover, E7 increases the ATPase activity of S4 and this same pathway was shown to be used by E7 in degrading pRb, with tumorigenic effect [135,157].

## 6. Concluding Remarks

The interplay between E6/E7 oncoproteins from various HPV types and the UPS has been shown to be one of the critical strategies used by these viruses to create an optimal environment in which they can successfully replicate. However, this process, which is generally highly regulated and controlled through the various stages of the viral life cycle, can in some instances be perturbed, initially causing an imbalance in cellular homeostasis, and ultimately resulting in the transformed cell phenotype. As can be seen from Figure 1 and Figure 2, by interacting with the components of the UPS, viral oncoproteins modulate numerous cellular functions, which are also necessary for initiation and/or maintenance of the transformed cellular phenotype. Interestingly, of the large number of α- and β-HPV types that infect humans, only a small proportion are actually associated with human malignancies. Intriguingly, as indicated in Table 1 and Table 2, it appears that E6/E7 oncoproteins from HR HPVs, which are associated with various human cancers at different anatomical sites, have been reported to interact with a vast number of the UPS components, including a number of ubiquitin ligases. In contrast, E6/E7 from β-HPVs, which are characterized as co-factors in causing skin cancers under specific conditions, interact with significantly fewer components of the UPS, while E6/E7 from LR types interact with only a few components of the UPS. All of this suggests that HR HPVs may be more efficiently adapted to evade the immune system and maintain persistent infection in the surrounding environment, than LR and β-HPV types, under permissive and regulated conditions. Consequently, when these conditions are disordered and the viral oncoproteins become unregulated, this is reflected in their interactions and modulation of the UPS, and can in turn lead to the formation of a more malignant phenotype, which is a hallmark of persistent HR HPV infections. Therefore, a better understanding of the interplay between the UPS and the viral oncoproteins is a pressing need for providing a more comprehensive network of the cellular pathways affected in the process of carcinogenesis in general, and HPV-induced carcinogenesis in particular. Furthermore, the development of novel strategies to block the viral perturbation of these UPS components should be one of the major aims in designing sorely-needed therapies against HPV-induced malignancies.

## Figures and Tables

**Table 1:** HPV E6 interactions with ubiquitin-proteasome system components.

Gene ID	Gene	Protein	Function	α-HPV		β-HPV	Method	Ref.
				LR	HR			
**580**	BARD1	Breast Cancer 1 Gene (BRCA1)- associated RING domain protein 1	Putative tumor suppressor gene mutated cancers. Homologous to BRCA1 RING motif and BRCT domain. BARD1/BRCA1 heterodimer is disrupted by tumorigenic amino acid substitutions in BRCA1. Heterodimer is required for BRCA1 tumor suppression and increases stability of both proteins.	−	+	?	Yeast-two hybrid, co-IP	Yim et al. (2007)
**672**	BRCA1	Breast Cancer type 1 susceptibility protein	Tumor suppressor. The E3 ubiquitin-protein ligase component of BARD1/BRCA1 heterodimer. BRCA1/BARD1 heterodimer coordinates DNA damage repair, ubiquitination and transcriptional regulation to maintain genomic stability.	?	+	?	IP, GST-pull down	Zhang et al. (2005)
**4850**	CNOT4	CCR4-NOT transcription complex subunit 4	E3 ubiquitin-protein ligase, promoting degradation of target proteins. Involved in JAK/STAT activation	−	−	+	IP, MS	White et al. (2012)
**1540**	CYLD	CYLD lysine 63 (K63) DUB	A lysine 63 (K63) deubiquitinase. Tumor suppressor negatively regulating NF-κB pathway. Ubiquitinated and degraded during Hypoxia-induced NF-κB activation to relieve its inhibition of NF-κB signaling cascade	?	+	?		An et al. (2008)
**8925**	HERC1	HECT and RLD domain containing E3 ubiquitin protein ligase family member 1	An E3 ubiquitin-protein ligase accepts ubiquitin from an E2 ubiquitin-conjugating enzyme and then transfers the ubiquitin to targeted substrates. Involved in membrane trafficking.	−	−	+	IP, proximity ligation in situ assay	Holloway et al. (2015)
**8924**	HERC2	HECT and RLD domain containing E3 ubiquitin- protein ligase 2	A putative HECT domain E3 ligase. Involved in protein trafficking and degradation pathways regulating ubiquitin-dependent retention of repair proteins on damaged chromosomes. Recruited to sites of DNA damage in response to ionizing radiation. Promotes DNA damage-induced formation of ‘Lys-63’-linked ubiquitin chains.	<+	+	<+	IP, MS	Vos et al. (2009); White et al. (2012)
**83737**	ITCH	E3 ubiquitin- protein ligase Itchy	An E3 ubiquitin-protein ligase which is involved in the control of inflammatory signaling pathways. An essential component of an ubiquitin-editing protein complex ensuring the transience of inflammatory signaling pathways by regulating ubiquitin-dependent signaling events. Involved in the cellular antiviral response.	?	+	?	GPCA	Poirson et al. (2017)
**23295**	MGRN1	Mahogunin ring finger 1	Has RING- E3 ubiquitin-protein ligase activity in vitro. Involved in regulation of endosome-to-lysosome trafficking. A negative regulator of hedgehog signaling.	+	+	+	GPCA, co-IP	Poirson et al. (2017)
**23024**	PDZRN3	PDZ domain containing ring finger 3	A member of the LNX (Ligand of Numb Protein-X) family of RING E3 ubiquitin- protein ligases. Required for vascular morphogenesis and differentiation of adipocytes, osteoblasts and myoblasts.	−	+	+	GST-pull down	Poirson et al. (2017); Thomas and Banks (2015)
**29951**	PDZRN4	PDZ domain containing ring finger 4	A member of the LNX family of RING E3 ubiquitin-protein ligases. A suppressor of cell proliferation in human liver cancer cell lines.	−	+	−	GPCA, co-IP	Poirson et al. (2017)
**5684**	PSMA3	Proteasome subunit alpha 3	Components of the 20S core proteasome complex involved in proteolysis of most cellular proteins. Associated with two 19S regulatory particles, form the 26S proteasome. Involved in ATP- dependent degradation of ubiquitinated proteins.	+	+	+	IP-MS/MS	White et al. (2012)
**5695**	PSMB7	Proteasome subunit beta 7		+	+	−	IP-MS/MS	White et al. (2012)
**5698**	PSMB9	Proteasome subunit beta 9		−	+	−	IP-MS/MS	White et al. (2012)
**5700**	PSMC1	Proteasome 26S subunit 4, ATPase 1	A component of the 26S proteasome belonging to the heterohexameric ring of AAA proteins (ATPases associated with diverse cellular activities). Unfolds ubiquitinated target proteins.	?	+	?	GST-pull down	Tomaić et al. (2013)
**5701**	PSMC2	Proteasome 26S subunit 7, ATPase 2	Components of the 26S proteasome.	+	+	+	IP-MS/MS	White et al. (2012); Tomaić et al. (2013)
**5702**	PSMC3	Proteasome 26S regulatory subunit 6A, ATPase 3		+	+	−	IP-MS/MS, GST-pull donw	White et al. (2012); Tomaić et al. (2013)
**5704**	PSMC4	Proteasome 26S subunit 6B, ATPase 4		+	+	−	IP-MS/MS	White et al. (2012)
**5705**	PSMC5	26S proteasome regulatory subunit 8		?	+	?	GST-pull down	Tomaić et al. (2013)
**5707**	PSMD1	26S proteasome non-ATPase regulatory subunit 1		+	+	+	IP-MS/MS	White et al. (2012)
**5708**	PSMD2	Proteasome 26S subunit 2, non-ATPase 2	Component of the 26S proteasome, binds to the intracellular domain of tumor necrosis factor type 1 receptor (TNFR1); the binding domain of TRAP1 and TRAP2 resides outside the death domain of TNFR1.	+	+	+	IP-MS/MS, GST-pull down	White et al. (2012); Tomaić et al. (2013)
**5710**	PSMD4	26S proteasome non-ATPase regulatory subunit 4	A major ubiquitin- accepting proteasome subunit. Involved in maintaining structural integrity of the 19S regulatory particle. Important in direct and indirect recognition of ubiquitinated substrates of 26S proteasome by interacting with polyubiquitinated proteins and directing them to the proteasome for degradation. A critical controlling factor in regulation of protein degradation at the proteasome.	?	+	?	IP-MS/MS	Tomaić et al. (2013)
**5709**	PSMD3	Proteasome 26S subunit 3, non-ATPase 3	Components of the 26S proteasome.	+	+	+	IP-MS/MS	White et al. (2012)
**9861**	PSMD6	Proteasome 26S subunit, non-ATPase 6		+	+	−	IP-MS/MS	White et al. (2012)
**5713**	PSMD7	Proteasome 26S subunit, non-ATPase 7		+	+	−	IP-MS/MS	White et al. (2012)
**5714**	PSMD8	Proteasome 26S subunit, non-ATPase 8		+	+	−	IP-MS/MS	White et al. (2012)
**5719**	PSMD13	26S proteasome non-ATPase regulatory subunit 13		+	+	+	IP-MS/MS	White et al. (2012)
**10213**	PSMD14	26S proteasome non-ATPase regulatory subunit 14		+	+	−	IP-MS/MS	White et al. (2012)
**23198**	PSME4	Proteasome activator subunit 4	A proteasome component that specifically recognizes and promotes ATP- and ubiquitin-independent degradation of acetylated core histones during DNA damage response to double-strand breaks.	−	+	−	IP-MS/MS	White et al. (2012)
**64320**	RNF25	Ring finger protein 25	A RING finger- dependent E3 ubiquitin- protein ligase that mediates ubiquitination and stimulates transcription mediated by NF-κB.	?	+	?	GPCA	Poirson et al. (2017)
**9810**	RNF40	Ring finger protein 40	A component of the RNF20/40 E3 ubiquitin- protein ligase complex; forms a H2B ubiquitin ligase complex in cooperation with the UBE2A or UBE2B. Supports maintenance of tumorigenic features and inflammatory signaling by promoting nuclear NF-κB activity.	?	+	?	GPCA	Poirson et al. (2017)
**85456**	TNKS1BP1	Tankyrase-1-binding protein	A subunit of the smaller 1-MDa core of Ccr4-Not complex. Ccr4-Not is an mRNA deadenylase and has a ubiquitin-protein ligase function.	<+	<+	+	IP-MS/MS	White et al. (2012)
**7188**	TRAF5	TNF receptor associated factor 5	Tumor necrosis factor receptor-associated factor (TRAF) protein family. An adapter protein and signal transducer linked to various signaling pathways by association with the receptor cytoplasmic domain and kinases. Involved in cytokine signaling and mediates activation of NF-κB and JNK. It is also involved in apoptosis.	?	+	?	GPCA	Poirson et al. (2017)
**7189**	TRAF6	TNF receptor- associated factor 6	An E3 ubiquitin-protein ligase that mediates the synthesis of ‘Lys-63’- linked-polyubiquitin chains conjugated to target proteins and ubiquitination of unanchored poly- ubiquitin chains. Induces activation of NF-κB and JUN. An adaptor protein and signal transducer linking TNFR proteins to different signaling pathways. Plays a role in signal transduction initiated via TNF receptor, IL-1 receptor and IL-17 receptor.	?	+	?	GPCA	Poirson et al. (2017)
**7706**	TRIM25	Tripartite motif containing 25	An E3 ubiquitin-protein ligase involved in innate immune defense against viruses. Crucially involved in the interferon response to viral infection.	+	+	+	co-IP	Chiang et al. (2018)
**10422**	UBAC1	UBA domain containing 1	A ubiquitin-protein ligase required for poly-ubiquitination and proteasome-mediated degradation of CDKN1B during G1 phase of the cell cycle. A non-catalytic subunit of the KPC Kip1 ubiquitination- promoting complex.	?	+	?	GPCA	Poirson et al. (2017)
**7337**	UBE3A	Ubiquitin- protein ligase E3A	An E3-HECT domain- containing ubiquitin- protein ligase. It promotes its own degradation in vivo. This imprinted gene is maternally expressed in the brain and biallelically expressed in other tissues. It plays an important role in regulation of the circadian clock and acts as a regulator of synaptic development.	<+	+	+	Yeast-two hybrid, co-crystal x-ray crystalography, co-IP	Poirson et al. (2017); Storey et al. (1998); Kao et al. (2000); Brimer et al., 2007; Thomas et al. (2013)
**23352**	UBR4	Ubiquitin- protein ligase E3 component n-recognin 4	An E3 ubiquitin-protein ligase, a component of the N-end rule pathway. Recognizes and binds to proteins bearing a specific N-terminal leading to ubiquitination and subsequent degradation. Forms meshwork structures involved in membrane morphogenesis and cytoskeletal organization and regulates integrin-mediated signaling.	−	−	+	IP	White et al. (2012); Thomas et al. (2013)
**51366**	UBR5	Ubiquitin- protein ligase E3 component n-recognin 5	Also known as EDD (E3 identified by Differential Display). A HECT domain-containing E3 ubiquitin-protein ligase component of the N-end rule pathway. Involved in coordinating the balance between cell cycle progression and differentiation. A regulator of DNA damage response, acts as a guard against excessive spreading of ubiquitinated chromatin at damaged chromosomes, as well as tumor suppressor. Frequently overexpressed in breast and ovarian cancer.	−	+	+	co-IP, MS	Tomaić et al. (2011); White et al. (2012)
**9958**	USP15	Ubiquitin specific peptidase 15	A deubiquitinating enzyme of the ubiquitin specific protease (USP) family. Plays a critical role in ubiquitin- dependent processes through polyubiquitin chain disassembly and hydrolysis of ubiquitin- substrate bonds.	−	+	+	Yeast-two hybrid, GPCA, IP	Vos et al. (2009); Poirson et al. (2017); Yaginuma et al. (2018); Chiang et al. (2018)
**64854**	USP46	Ubiquitin carboxyl- terminal hydrolase 46	A deubiquitinating enzyme that plays a role in behavior by regulating GABA action. Has little intrinsic deubiquitinating activity and requires interaction with regulator of deubiquitinating complexes WDR48 (WD repeat-containing protein 48) for high activity.	−	+	?	co-IP	Kiran et al. (2018)
**7428**	VHL	Von Hippel-Lindau tumor suppressor	VHL is a component of the protein complex that includes elongin C, elongin B, and cullin-2. An E3 ubiquitin-protein ligase involved in the ubiquitination and degradation of hypoxia- inducible factor (HIF), a transcription factor crucial to oxygen-related gene expression.. HPV16 E6 promotes hypoxia- induced Warburg effect through blocking the association of HIF-1α and VHL	?	+	?	GPCA	Poirson et al. (2017); Guo et al. (2014)
**331**	XIAP	X-linked inhibitor of apoptosis	A multi-functional protein that regulates apoptosis, modulates inflammatory signaling and immunity, cell proliferation, cell invasion and metastasis. An E3 ubiquitin-protein ligase regulating NF-κB signaling and other target. An important regulator of innate immune signaling via regulation of Nod-like receptors (NLRs).	?	+	?	GPCA	Poirson et al. (2017)

+ confirmed to interact; +, < confirmed interactions with lower affinity compared to hR-HPV E6; - interactions not detected; ? interactions not tested. Co-IP: co-immunoprecipitation; GPCA: Gaussia princeps luciferase protein complementation assay, IP-MS/MS: immunoprecipitation-mass spectrometry, IP: immunoprecipitation.

**Table 2:** HPV E7 interactions with ubiquitin-proteasome system components.

Gene ID	Gene	Protein	Function	α-HPV		β-HPV	Method	Ref.
				LR	HR			
**8454**	CUL1	Cullin-1	A core component of cullin-RING-based SCF E3 ubiquitin- protein ligase complex, which mediates ubiquitination and proteolysis of E7.	+	+	?	IP	Münger et al. (1989); Boyer et al. (1996); Oh et al. (2004)
**8453**	CUL2	Cullin-2	A core component of cullin-RING-based ECS E3 ubiquitin- protein ligase complex. Stabilizes APOBEC3A. Required for E7-induced degradation of pRB contributing to cell transformation by dysregulating G1/S cell cycle checkpoints	−	+	-	co-IP	Huh et al. (2007); Narisawa-Saito and Kiyono. (2007); White et al. (2012); Xu et al. (2016); Westrich et al. (2018)
**8452**	CUL3	Cullin-3	A core component of cullin-RING-based BCR E3 ubiquitin- protein ligase complexes, which mediate the ubiquitination and proteasomal degradation of target proteins.	+	+	?	co-IP	White et al. (2012); Poirson et al. (2017)
**90379**	DCAF15	DDB1 and CUL4 associated factor 15	May be involved in ubiquitination and degradation through a DBB1-CUL4 E3 protein-ubiquitin ligase.	−	+	−	GPCA, co-IP	Poirson et al. (2017)
**253980**	KCTD13	Potassium channel tetramer- ization domain 13	A substrate-specific adapter of a BCR E3 ubiquitin-protein ligase complex required for synaptic transmission.	+	+	+	GPCA, co-IP	Chen et al. (2009); Poirson et al. (2017)
**112939**	NACC1	Nucleus accumbens- associated protein 1	A transcriptional repressor and transcriptional corepressor in neuronal cells through recruitment of HDAC3 and HDAC4 Required to recruit the proteasome from the nucleus to the cytoplasm and dendritic spines.	?	+	?	GPCA	Poirson et al. (2017)
**9148**	NEURL1	Neuralized E3 ubiquitin- protein ligase 1	An E3 ubiquitin- protein ligase that activates in vitro ubiquitination of JAG1, inhibiting malignant cell transformation of medulloblastoma cells through the Notch pathway.	+	+	+	GPCA, co-IP	Poirson et al. (2017)
**5700**	PSMC1	26S proteasome regulatory subunit 4	A component of the 26S proteasome.	−	+	?	IP	Berezutskaya and Bagchi (1997); Ben-Saadon et al. (2004)
**84282**	RNF135	ring finger protein 135	An E2-dependent E3 ubiquitin-protein ligase, involved in innate immune defense against viruses.	+, <	+	−	GPCA, co-IP	Poirson et al. (2017)
**57630**	SH3RF1	SH3 domain containing ring finger 1	Has an E3 ubiquitin-protein ligase activity. In the absence of an external substrate, it can catalyze self-ubiquitination.	+, <	+	?	GPCA	Poirson et al. (2017)
**92799**	SHKBP1	SH3KBP1- binding protein 1	SHKBP1 inhibits CBL-SH3KBP1 complex mediated downregulation of EGFR signaling by sequestration of SH3KBP1.	+	+	+	GPCA, co-IP	Poirson et al. (2017)
**7126**	TNFAIP1	TNF alpha induced protein 1	A substrate-specific adapter of a BCR E3 ubiquitin-protein ligase complex that mediates the ubiquitination and proteasomal degradation of RhoA, thereby regulating the actin cytoskeleton and cell migration. HPV-16 E7 can modulate the responses of its natural host cell to the closely related cytokines TNF-α.	+	+	+	GPCA, co-IP	Basile et al. (2001); Poirson et al. (2017)
**7186**	TRAF2	TNF receptor associated factor 2	Regulates the activation of NF-κB and JNK and plays a central role in the regulation of cell survival and apoptosis. An essential constituent of several E3 ubiquitin-protein ligases. HPV16 E6/E7 switch cells from apoptotic to proliferative fates under TWEAK/Fn14 interaction, possibly by favoring Ras and TRAF2 activation and modulating TNF receptor expression.	−	+	+	GPCA, co-IP	Cheng et al. (2015); Poirson et al. (2017)
**7187**	TRAF3	TNF receptor associated factor 3	Regulates pathways leading to activation of NF-κB and MAP kinases, and plays a central role in the regulation of B-cell survival. An essential constituent of several E3 ubiquitin-protein ligase complexes. Overexpression of TRAF3 enhances p53 and pRb expression	?	+	?	GPCA	Poirson et al. (2017); Zhang et al. (2018)
**9618**	TRAF4	TNF receptor associated factor 4	An adaptor protein and signal transducer linking members of the TNFR family to different signaling pathways. Plays a role in the activation of NF-κB and JNK, and in the regulation of cell survival and apoptosis. May interact selectively and non-covalently with E3 ubiquitin- protein ligase enzymes.	?	+	?	GPCA	Poirson et al. (2017)
**7188**	TRAF5	TNF receptor associated factor 5	An adaptor protein and signal transducer linking members of the TNFR family to different signaling pathways. May interact selectively and non-covalently with E3 ubiquitin-protein ligase enzymes.	+	+	+	GPCA, co-IP	Poirson et al. (2017)
**10346**	TRIM22	tripartite motif containing 22	An interferon- induced antiviral protein involved in innate immunity. May have E3 ubiquitin-protein ligase activity. Activated by integration of E6/E7 genes.	?	+	?	GPCA	Pett et al. (2006); Poirson et al. (2017)
**22954**	TRIM32	tripartite motif containing 32	An E3 ubiquitin-protein ligase. It ubiquitinates DTNBP1 and promotes its degradation.	+	+	+	GPCA, co-IP	Poirson et al. (2017)
**493829**	TRIM72	tripartite motif containing 72	A muscle-specific protein that plays a central role in cell membrane repair by nucleating the assembly of the repair machinery at injury sites. May be involved in proteasome- mediated, ubiquitin-dependent protein catabolic processes.	−	+	+	GPCA, co-IP	Poirson et al. (2017)
**114088**	TRIM9	tripartite motif containing 9	An E3 ubiquitin- protein ligase, which self-ubiquitinates in cooperation with E2 enzyme UBE2D2/UBC4. Serves as a targeting signal for proteasomal degradation.	?	+	?	GPCA	Poirson et al. (2017)
**7314**	UBB	ubiquitin B	Targets cellular proteins for degradation by the 26S proteasome. Involved in the maintenance of chromatin structure, the regulation of gene expression, and the stress response.	?	+	?	GPCA	Poirson et al. (2017)
**7319**	UBE2A	Ubiquitin- conjugating enzyme E2A	Accepts ubiquitin from the E1 complex. In association with the E3 enzyme, UBE2A plays a role in transcription regulation by catalyzing the ubiquitination of histone H2B.	+	+	?	GPCA	Poirson et al. (2017)
**7332**	UBE2L3	Ubiquitin- conjugating enzyme E2L3	Specifically acts with HECT-type and RBR family E3 ubiquitin- protein ligases. Accepts ubiquitin from the E1 complex and catalyzes its covalent attachment to other proteins including E7.	?	+	?	IP	Reinstein et al. (2000)
**23352**	UBR4	Ubiquitin- protein ligase E3 component n-recognin 4	Also known as p600. An E3 ubiquitin- protein ligase that recognizes proteins with specific destabilized N-terminal residues, leading to their ubiquitination and degradation. May mediate some pRB- independent transforming activities of HPV-16 E7, but is not sufficient for cellular transformation as interactions were also found with low-risk HPV E7 oncoproteins. It is speculated that UBR4 could potentially play a role in viral replication.	+	+	+	IP	Huh et al. (2005); De Masi et al. (2005); White et al. (2012)
**8237**	USP11	Ubiquitin carboxyl- terminal hydrolase 11	Inhibits degradation of target proteins by the proteasome. Plays a role in the regulation of pathways leading to NF-κ-B activation. Augments HPV-16E7 activity in modulating downstream target genes, such as pRb, Bcl-2, and Cdc-2, suggesting that this interaction may contribute to cell transformation by HPV-16E7.	?	+	?	IP	Lin et al. (2008); Poirson et al. (2017)
**83844**	USP26	Ubiquitin carboxyl- terminal hydrolase 26	Involved in the ubiquitin-dependent proteolytic pathway in conjunction with the 26S proteasome.	+	+	+	GPCA, co-IP	Poirson et al. (2017)
**57663**	USP29	Ubiquitin carboxyl- terminal hydrolase 29	A thiol-dependent hydrolyser of ester, thioester, amide, peptide and isopeptide bonds formed by the C-terminal Gly of ubiquitin.	−	+	−	GPCA, co-IP	Poirson et al. (2017)
**23032**	USP33	Ubiquitin carboxyl- terminal hydrolase 33	A deubiquitinating enzyme involved in centrosome duplication, cell migration and beta-2 adrenergic receptor/ADRB2 recycling.	+	+	+	GPCA, co-IP	Poirson et al. (2017)
**90850**	ZNF598	Zinc finger protein 598	An E3 ubiquitin- protein ligase required for terminal stalling of ribosomes during translation of poly(A) sequences by mediating ubiquitination of 40S ribosomal protein.	?	+	?	GPCA	Poirson et al. (2017)

+ confirmed to interact; +, < confirmed interactions with lower affinity compared to HR-HPV E7; - interactions not detected; ? interactions not tested. Co-IP: co-immunoprecipitation; GPCA: *Gaussia princeps luciferase* protein complementation assay, IP-MS/MS: immunoprecipitation-mass spectrometry, IP: immunoprecipitation.

## References

[1] International Agency for Cancer Research (2005). IARC Monographs 100B—Human Papillomaviruses 2012.

[2] King A.M.Q., Adams M.J., Carstens E.B., Lefkowitz E.J. (2005). Virus Taxonomy: Classification and Nomenclature of Viruses. Virus Taxon..

[3] López-Bueno A., Mavian C., Labella A.M., Castro D., Borrego J.J., Alcami A., Alejo A. (2016). Concurrence of Iridovirus, Polyomavirus, and a Unique Member of a New Group of Fish Papillomaviruses in Lymphocystis Disease-Affected Gilthead Sea Bream. J. Virol..

[4] de Villiers E.-M., Burk R.D., Bernard H.-U., Chen Z., van Doorslaer K., zur Hausen H. (2010). Classification of papillomaviruses (PVs) based on 189 PV types and proposal of taxonomic amendments. Virology.

[5] zur Hausen H. (2002). Papillomaviruses and cancer: From basic studies to clinical application. Nat. Rev. Cancer.

[6] Boda D., Docea A.O., Calina D., Ilie M.A., Caruntu C., Zurac S., Mamoulakis C. (2018). Human papilloma virus: Apprehending the link with carcinogenesis and unveiling new research avenues (Review). Int. J. Oncol..

[7] Bouvard V., Baan R., Straif K., Grosse Y., Secretan B., El Ghissassi F., Cogliano V. (2009). A review of human carcinogens-Part B: Biological agents. Lancet Oncol..

[8] De Villiers E.M., Fauquet C., Broker T.R., Bernard H.U., Zur Hausen H. (2004). Classification of papillomaviruses. Virology.

[9] Burd E. (2003). Human papillomavirus and cervical cancerBurd E (2003) Human papillomavirus and cervical cancer. Clin. Microbiol. Rev..

[10] Thomas M., Narayan N., Pim D., Tomaić V., Massimi P., Nagasaka K., Banks L. (2008). Human papillomaviruses, cervical cancer and cell polarity. Oncogene.

[11] Syrjänen K.J., Pyrhönen S., Syrjänen S.M., Lamberg M.A. (1985). Immunohistochemical demonstration of human papilloma virus (HPV) antigens in oral squamous cell lesions. Fed. Proc..

[12] Spence T., Bruce J., Yip K.W., Liu F.F. (2016). HPV associated head and neck cancer. Cancers.

[13] Chaturvedi A.K., Engels E.A., Pfeiffer R.M., Hernandez B.Y., Xiao W., Kim E., Liu L. (2011). Human papillomavirus and rising oropharyngeal cancer incidence in the United States. J. Clin. Oncol..

[14] Combes J.-D., Chen A.A., Franceschi S. (2014). Prevalence of human papillomavirus in cancer of the oropharynx by gender. Cancer Epidemiol. Biomark. Prev..

[15] Tomaić V. (2016). Functional Roles of E6 and E7 Oncoproteins in HPV-Induced Malignancies at Diverse Anatomical Sites. Cancers.

[16] Tommasino M. (2017). The biology of beta human papillomaviruses. Virus Res..

[17] Egawa N., Doorbar J. (2017). The low-risk papillomaviruses. Virus Res..

[18] Howley P.M., Pfister H.J. (2015). Beta genus papillomaviruses and skin cancer. Virology.

[19] Strickley J.D., Messerschmidt J.L., Awad M.E., Li T., Hasegawa T., Ha D.T., Nazarian R.M. (2019). Immunity to commensal papillomaviruses protects against skin cancer. Nature.

[20] Doorbar J. (2005). The papillomavirus life cycle. J. Clin. Virol..

[21] Moody C.A., Laimins L.A. (2010). Human papillomavirus oncoproteins: Pathways to transformation. Nat. Rev. Cancer.

[22] Morgan E.L., Wasson C.W., Hanson L., Kealy D., Pentland I., McGuire V., Roberts S. (2018). STAT3 activation by E6 is essential for the differentiation-dependent HPV18 life cycle. PLoS Pathog..

[23] Snijders P.J.F., Steenbergen R.D.M., Heideman D.A.M., Meijer C.J.L.M. (2006). HPV-mediated cervical carcinogenesis: Concepts and clinical implications. J. Pathol..

[24] Steben M., Duarte-Franco E. (2007). Human papillomavirus infection: Epidemiology and pathophysiology. Gynecol. Oncol..

[25] Boxman I.L.A., Berkhout R.J.M., HCMulder L., Wolkers M.C., Bavinck J.N.B., Vermeer B.J., ter Schegget J. (1997). Detection of human papillomavirus DNA in plucked hairs from renal transplant recipients and healthy volunteers. J. Investig. Dermatol..

[26] Feltkamp M.C.W., de Koning M.N.C., Bavinck J.N.B., ter Schegget J. (2008). Betapapillomaviruses: Innocent bystanders or causes of skin cancer. J. Clin. Virol..

[27] Caldeira S., Zehbe I., Accardi R., Malanchi I., Dong W., Giarrè M., Tommasino M. (2003). The E6 and E7 Proteins of the Cutaneous Human Papillomavirus Type 38 Display Transforming Properties. J. Virol..

[28] Viarisio D., Mueller-Decker K., Kloz U., Aengeneyndt B., Kopp-Schneider A., Gröne H.-J., Tommasino M. (2011). E6 and E7 from beta HPV38 cooperate with ultraviolet light in the development of actinic keratosis-like lesions and squamous cell carcinoma in mice. PLoS Pathog..

[29] Quint K.D., Genders R.E., de Koning M.N.C., Borgogna C., Gariglio M., Bouwes Bavinck J.N., Feltkamp M.C. (2015). Human Beta-papillomavirus infection and keratinocyte carcinomas. J. Pathol..

[30] Meyers J.M., Munger K. (2014). The viral etiology of skin cancer. J. Investig. Dermatol..

[31] Viarisio D., Müller-Decker K., Accardi R., Robitaille A., Dürst M., Beer K., Voegele C. (2018). Beta HPV38 oncoproteins act with a hit-and-run mechanism in ultraviolet radiation-induced skin carcinogenesis in mice. PLoS Pathog..

[32] Hershko A., Ciechanover A. (1997). The ubiquitin system. Annu. Rev. Biochem..

[33] Ciechanover A. (2005). Intracellular protein degradation: From a Vague Idea, through the lysosome and the ubiquitin-proteasome system, and onto human diseases and drug targeting (Nobel Lecture). Angew. Chem. Int. Ed..

[34] Tanaka K. (2009). The proteasome: Overview of structure and functions. Proc. Jpn. Acad. Ser. B.

[35] Rousseau A., Bertolotti A. (2018). Regulation of proteasome assembly and activity in health and disease. Nat. Rev. Mol. Cell Biol..

[36] Saeki Y. (2017). Ubiquitin recognition by the proteasome. J. Biochem..

[37] Finley D. (2009). Recognition and processing of ubiquitin-protein conjugates by the proteasome. Annu. Rev. Biochem..

[38] Poirson J., Biquand E., Straub M.-L., Cassonnet P., Nominé Y., Jones L., Demeret C. (2017). Mapping the interactome of HPV E6 and E7 oncoproteins with the ubiquitin-proteasome system. FEBS J..

[39] Ingham R.J., Gish G., Pawson T. (2004). The Nedd4 family of E3 ubiquitin ligases: Functional diversity within a common modular architecture. Oncogene.

[40] Li W., Bengtson M.H., Ulbrich A., Matsuda A., Reddy V.A., Orth A., Joazeiro C.A. (2008). Genome-wide and functional annotation of human E3 ubiquitin ligases identifies MULAN, a mitochondrial E3 that regulates the organelle’s dynamics and signaling. PLoS ONE.

[41] Li W., Ye Y. (2008). Polyubiquitin chains: Functions, structures, and mechanisms. Cell. Mol. Life Sci..

[42] Rotin D., Kumar S. (2009). Physiological functions of the HECT family of ubiquitin ligases. Nat. Rev. Mol. Cell Biol..

[43] Wing S.S. (2003). Deubiquitinating enzymes-the importance of driving in reverse along the ubiquitin-proteasome pathway. Int. J. Biochem. Cell Biol..

[44] Tomaić V., Banks L. (2015). Angelman syndrome-associated ubiquitin ligase UBE3A/E6AP mutants interfere with the proteolytic activity of the proteasome. Cell Death Dis..

[45] Lou Z., Wang S. (2014). E3 ubiquitin ligases and human papillomavirus-induced carcinogenesis. J. Int. Med. Res..

[46] Vos R.M., Altreuter J., White E.A., Howley P.M. (2009). The Ubiquitin-Specific Peptidase USP15 Regulates Human Papillomavirus Type 16 E6 Protein Stability. J. Virol..

[47] Tomaić V., Pim D., Banks L. (2009). The stability of the human papillomavirus E6 oncoprotein is E6AP dependent. Virology.

[48] Scheffner M., Werness B.A., Huibregtse J.M., Levine A.J., Howley P.M. (1990). The E6 oncoprotein encoded by human papillomavirus types 16 and 18 promotes the degradation of p53. Cell.

[49] Helt A.-M., Funk J.O., Galloway D.A. (2002). Inactivation of both the Retinoblastoma Tumor Suppressor and p21 by the Human Papillomavirus Type 16 E7 Oncoprotein Is Necessary To Inhibit Cell Cycle Arrest in Human Epithelial Cells. J. Virol..

[50] Münger K., Werness B.A., Dyson N., Phelps W.C., Harlow E., Howley P.M. (1989). Complex formation of human papillomavirus E7 proteins with the retinoblastoma tumor suppressor gene product. EMBO J..

[51] Werness B.A., Levine A.J., Howley P.M. (1990). Association of human papillomavirus types 16 and 18 E6 proteins with p53. Science.

[52] Pietsch E.C., Murphy M.E. (2008). Low risk HPV-E6 traps p53 in the cytoplasm and induces p53-dependent apoptosis. Cancer Biol. Ther..

[53] Oh S.T., Longworth M.S., Laimins L.A. (2004). Roles of the E6 and E7 Proteins in the Life Cycle of Low-Risk Human Papillomavirus Type 11. J. Virol..

[54] Crook T., Tidy J.A., Vousden K.H. (1991). Degradation of p53 can be targeted by HPV E6 sequences distinct from those required for p53 binding and trans-activation. Cell.

[55] Lechner M.S., Laimins L.A. (1994). Inhibition of p53 DNA binding by human papillomavirus E6 proteins. J. Virol..

[56] White E.A., Walther J., Javanbakht H., Howley P.M. (2014). Genus beta human papillomavirus E6 proteins vary in their effects on the transactivation of p53 target genes. J. Virol..

[57] White E.A., Sowa M.E., Tan M.J.A., Jeudy S., Hayes S.D., Santha S., Münger K., Harper J.W., Howley P.M. (2012). Systematic identification of interactions between host cell proteins and E7 oncoproteins from diverse human papillomaviruses. Proc. Natl. Acad. Sci. USA.

[58] Huh K., Zhou X., Hayakawa H., Cho J.-Y., Libermann T.A., Jin J., Harper J.W., Munger K. (2007). Human Papillomavirus Type 16 E7 Oncoprotein Associates with the Cullin 2 Ubiquitin Ligase Complex, Which Contributes to Degradation of the Retinoblastoma Tumor Suppressor. J. Virol..

[59] Szalmás A., Tomaić V., Basukala O., Massimi P., Mittal S., Kónya J., Banks L. (2017). The PTPN14 Tumor Suppressor Is a Degradation Target of Human Papillomavirus E7. J. Virol..

[60] Holloway A., Simmonds M., Azad A., Fox J.L., Storey A. (2015). Resistance to UV-induced apoptosis by β-HPV5 E6 involves targeting of activated BAK for proteolysis by recruitment of the HERC1 ubiquitin ligase. Int. J. Cancer.

[61] Bennett Saidu N.E., Filić V., Thomas M., Sarabia-Vega V., Ðukić A., Miljković F., Banks L., Tomaić V. (2019). PDZ Domain-Containing Protein NHERF-2 is a Novel Target of Human Papillomavirus type 16 (HPV-16) and HPV-18. J. Virol..

[62] Huh K.-W., DeMasi J., Ogawa H., Nakatani Y., Howley P.M., Münger K. (2005). Association of the human papillomavirus type 16 E7 oncoprotein with the 600-kDa retinoblastoma protein-associated factor, p600. Proc. Natl. Acad. Sci. USA.

[63] DeMasi J., Huh K.-W., Nakatani Y., Münger K., Howley P.M. (2005). Bovine papillomavirus E7 transformation function correlates with cellular p600 protein binding. Proc. Natl. Acad. Sci. USA.

[64] Grm H.S., Banks L. (2004). Degradation of hDlg and MAGIs by human papillomavirus E6 is E6-AP-independent. J. Gen. Virol..

[65] Cole S.T., Danos O. (1987). Nucleotide sequence and comparative analysis of the human papillomavirus type 18 genome. Phylogeny of papillomaviruses and repeated structure of the E6 and E7 gene products. J. Mol. Biol..

[66] Barbosa M.S., Wettstein F.O. (1987). Transcription of the cottontail rabbit papillomavirus early region and identification of two E6 polypeptides in COS-7 cells. J. Virol..

[67] Martinez-Zapien D., Ruiz F.X., Poirson J., Mitschler A., Ramirez J., Forster A., Cousido-Siah A., Masson M., Pol S.V., Podjarny A. (2016). Structure of the E6/E6AP/p53 complex required for HPV-mediated degradation of p53. Nature.

[68] Kanda T., Watanabe S., Zanma S., Sato H., Furuno A., Yoshiike K. (1991). Human papillomavirus type 16 E6 proteins with glycine substitution for cysteine in the metal-binding motif. Virology.

[69] Sherman L., Schlegel R. (1996). Serum- and calcium-induced differentiation of human keratinocytes is inhibited by the E6 oncoprotein of human papillomavirus type 16. J. Virol..

[70] Nominé Y., Masson M., Charbonnier S., Zanier K., Ristriani T., Deryckère F., Sibler A.P., Desplancq D., Atkinson R.A., Weiss E. (2006). Structural and functional analysis of E6 oncoprotein: Insights in the molecular pathways of human papillomavirus-mediated pathogenesis. Mol. Cell..

[71] Zanier K., Ruhlmann C., Melin F., Masson M., Ould M’hamed Ould Sidi A., Bernard X., Fischer B., Brino L., Ristriani T., Rybin V. (2010). E6 proteins from diverse papillomaviruses self-associate both in vitro and in vivo. J Mol Biol.

[72] Zanier K., Charbonnier S., Sidi A.O.M.O., McEwen A.G., Ferrario M.G., Poussin-Courmontagne P., Cura V., Brimer N., Babah K.O., Ansari T. (2013). Structural basis for hijacking of cellular LxxLL motifs by papillomavirus E6 oncoproteins. Science.

[73] Pim D., Banks L. (2010). Interaction of viral oncoproteins with cellular target molecules: Infection with high-risk vs low-risk human papillomaviruses. APMIS.

[74] Zhang Y., Dasgupta J., Ma R.Z., Banks L., Thomas M., Chen X.S. (2007). Structures of a human papillomavirus (HPV) E6 polypeptide bound to MAGUK proteins: Mechanisms of targeting tumor suppressors by a high-risk HPV oncoprotein. J. Virol..

[75] Huibregtse J.M., Scheffner M., Howley P.M. (1993). Localization of the E6-AP regions that direct human papillomavirus E6 binding, association with p53, and ubiquitination of associated proteins. Mol. Cell. Biol..

[76] Elston R.C., Napthine S., Doorbar J. (1998). The identification of a conserved binding motif within human papillomavirus type 16 E6 binding peptides, E6AP and E6BP. J. Gen. Virol..

[77] White E.A., Kramer R.E., Tan M.J.A., Hayes S.D., Harper J.W., Howley P.M. (2012). Comprehensive Analysis of Host Cellular Interactions with Human Papillomavirus E6 Proteins Identifies New E6 Binding Partners and Reflects Viral Diversity. J. Virol..

[78] Brimer N., Lyons C., Wallberg A.E., Vande Pol S.B. (2012). Cutaneous papillomavirus E6 oncoproteins associate with MAML1 to repress transactivation and NOTCH signaling. Oncogene.

[79] Tan M.J.A., White E.A., Sowa M.E., Harper J.W., Aster J.C., Howley P.M. (2012). Cutaneous β-human papillomavirus E6 proteins bind Mastermind-like coactivators and repress Notch signaling. Proc. Natl. Acad. Sci. USA.

[80] Meyers J.M., Spangle J.M., Munger K. (2013). The human papillomavirus type 8 E6 protein interferes with NOTCH activation during keratinocyte differentiation. J. Virol..

[81] Brimer N., Drews C.M., Vande Pol S.B. (2017). Association of papillomavirus E6 proteins with either MAML1 or E6AP clusters E6 proteins by structure, function, and evolutionary relatedness. PLoS Pathog..

[82] Thomas M., Tomaić V., Pim D., Myers M.P., Tommasino M., Banks L. (2013). Interactions between E6AP and E6 proteins from alpha and beta HPV types. Virology.

[83] Brimer N., Lyons C., Vande Pol S.B. (2007). Association of E6AP (UBE3A) with human papillomavirus type 11 E6 protein. Virology.

[84] Scheffner M., Huibregtse J.M., Vierstra R.D., Howley P.M. (1993). The HPV-16 E6 and E6-AP complex functions as a ubiquitin-protein ligase in the ubiquitination of p53. Cell.

[85] Huibregtse J.M., Scheffner M., Howley P.M. (1993). Cloning and expression of the cDNA for E6-AP, a protein that mediates the interaction of the human papillomavirus E6 oncoprotein with p53. Mol. Cell. Biol..

[86] Talis A.L., Huibregtse J.M., Howley P.M. (1998). The role of E6AP in the regulation of p53 protein levels in human papillomavirus (HPV)-positive and HPV-negative cells. J. Biol. Chem..

[87] Kelley M.L., Keiger K.E., Lee C.J., Huibregtse J.M. (2005). The global transcriptional effects of the human papillomavirus E6 protein in cervical carcinoma cell lines are mediated by the E6AP ubiquitin ligase. J. Virol..

[88] Storey A., Thomas M., Kalita A., Harwood C., Gardiol D., Mantovani F., Breuer J., Leigh I.M., Matlashewski G., Banks L. (1998). Role of a p53 polymorphism in the development of human papillomavirus-associated cancer. Nature.

[89] Ji Y., Walkowicz M.J., Buiting K., Johnson D.K., Tarvin R.E., Rinchik E.M., Horsthemke B., Stubbs L., Nicholls R.D. (1999). The ancestral gene for transcribed, low-copy repeats in the Prader-Willi/Angelman region encodes a large protein implicated in protein trafficking, which is deficient in mice with neuromuscular and spermiogenic abnormalities. Hum. Mol. Genet..

[90] Martínez-Noël G., Galligan J.T., Sowa M.E., Arndt V., Overton T.M., Harper J.W., Howley P.M. (2012). Identification and Proteomic Analysis of Distinct UBE3A/E6AP Protein Complexes. Mol. Cell. Biol..

[91] Kühnle S., Kogel U., Glockzin S., Marquardt A., Ciechanover A., Matentzoglu K., Scheffner M. (2011). Physical and Functional Interaction of the HECT Ubiquitin-protein Ligases E6AP and HERC2. J. Biol. Chem..

[92] Callaghan M.J., Russell A.J., Woollatt E., Sutherland G.R., Sutherland R.L., Watts C.K. (1998). Identification of a human HECT family protein with homology to the Drosophila tumor suppressor gene hyperplastic discs. Oncogene.

[93] Clancy J.L., Henderson M.J., Russell A.J., Anderson D.W., Bova R.J., Campbell I.G., Choong D.Y., Macdonald G.A., Mann G.J., Nolan T. (2003). EDD, the human orthologue of the hyperplastic discs tumour suppressor gene, is amplified and overexpressed in cancer. Oncogene.

[94] Tomaić V., Pim D., Thomas M., Massimi P., Myers M.P., Banks L. (2011). Regulation of the Human Papillomavirus Type 18 E6/E6AP Ubiquitin Ligase Complex by the HECT Domain-Containing Protein EDD. J. Virol..

[95] Subbaiah V.K., Zhang Y., Rajagopalan D., Abdullah L.N., Yeo-Teh N.S.L., Tomaić V., Banks L., Myers M.P., Chow E.K., Jha S. (2016). E3 ligase EDD1/UBR5 is utilized by the HPV E6 oncogene to destabilize tumor suppressor TIP60. Oncogene.

[96] Hayman T.J., Hsu A.C., Kolesnik T.B., Dagley L.F., Willemsen J., Tate M.D., Baker P.J., Kershaw N.J., Kedzierski L., Webb A.I. RIPLET, and not TRIM25, is required for endogenous RIG-I-dependent antiviral responses. Immunol. Cell Biol..

[97] Oshiumi H., Miyashita M., Matsumoto M., Seya T. (2013). A distinct role of Riplet-mediated K63-Linked polyubiquitination of the RIG-I repressor domain in human antiviral innate immune responses. PLoS Pathog..

[98] Chiang C., Pauli E.-K., Biryukov J., Feister K.F., Meng M., White E.A. (2018). The Human Papillomavirus E6 Oncoprotein Targets USP15 and TRIM25 To Suppress RIG-I-Mediated Innate Immune Signaling. J. Virol..

[99] Wu L.C., Wang Z.W., Tsan J.T., Spillman M.A., Phung A., Xu X.L., Yang M.C.W., Hwang L.Y., Bowcock A.M., Baer R. (1996). Identification of a RING protein that can interact in vivo with the BRCA1 gene product. Nat. Genet..

[100] Morris J.R., Keep N.H., Solomon E. (2002). Identification of residues required for the interaction of BARD1 with BRCA1. J. Biol. Chem..

[101] Yim E.-K., Lee K.-H., Myeong J., Tong S.-Y., Um S.-J., Park J.-S. (2007). Novel interaction between HPV E6 and BARD1 (BRCA1-associated ring domain 1) and its biologic roles. DNA Cell Biol..

[102] Zhang Y., Fan S., Meng Q., Ma Y., Katiyar P., Schlegel R., Rosen E.M. (2005). BRCA1 interaction with human papillomavirus oncoproteins. J. Biol. Chem..

[103] Thomas M., Banks L. (2015). PDZRN3/LNX3 is a novel target of human papillomavirus type 16 (HPV-16) and HPV-18 E6. J. Virol..

[104] Guo Y., Meng X., Ma J., Zheng Y., Wang Q., Wang Y., Shang H. (2014). Human papillomavirus 16 E6 contributes HIF-1α induced Warburg effect by attenuating the VHL-HIF-1α interaction. Int. J. Mol. Sci..

[105] Albert T.K., Hanzawa H., Legtenberg Y.I.A., de Ruwe M.J., van den Heuvel F.A.J., Collart M.A., Boelens R., Timmers H.T.M. (2002). Identification of a ubiquitin–protein ligase subunit within the CCR4–NOT transcription repressor complex. EMBO J..

[106] Schaper I.D., Marcuzzi G.P., Weissenborn S.J., Kasper H.U., Dries V., Smyth N., Fuchs P., Pfister H. (2005). Development of skin tumors in mice transgenic for early genes of human papillomavirus type 8. Cancer Res..

[107] Hatterschide J., Bohidar A.E., Grace M., Nulton T.J., Kim H.W., Windle B., Morgan I.M., Munger K., White E.A. (2019). PTPN14 degradation by high-risk human papillomavirus E7 limits keratinocyte differentiation and contributes to HPV-mediated oncogenesis. Proc. Natl. Acad. Sci. USA.

[108] White E.A., Münger K., Howley P.M. (2016). High-Risk Human Papillomavirus E7 Proteins Target PTPN14 for Degradation. MBio.

[109] An J., Mo D., Liu H., Veena M.S., Srivatsan E.S., Massoumi R., Rettig M.B. (2008). Inactivation of the CYLD deubiquitinase by HPV E6 mediates hypoxia-induced NF-kappaB activation. Cancer Cell.

[110] Kiran S., Dar A., Singh S.K., Lee K.Y., Dutta A. (2018). The Deubiquitinase USP46 Is Essential for Proliferation and Tumor Growth of HPV-Transformed Cancers. Mol. Cell.

[111] Tomaić V., Ganti K., Pim D., Bauer C., Blattner C., Banks L. (2013). Interaction of HPV E6 oncoproteins with specific proteasomal subunits. Virology.

[112] Rozenblatt-Rosen O., Deo R.C., Padi M., Adelmant G., Calderwood M.A., Rolland T., Byrdsong D., Correll M., Fan C., Feltkamp M.C. (2012). Interpreting cancer genomes using systematic host network perturbations by tumour virus proteins. Nature.

[113] Scanlon T.C., Gottlieb B., Durcan T.M., Fon E.A., Beitel L.K., Trifiro M.A. (2009). Isolation of human proteasomes and putative proteasome-interacting proteins using a novel affinity chromatography method. Exp. Cell Res..

[114] Besche H.C., Haas W., Gygi S.P., Goldberg A.L. (2009). Isolation of mammalian 26S proteasomes and p97/VCP complexes using the ubiquitin-like domain from HHR23B reveals novel proteasome-associated proteins. Biochemistry.

[115] Kleijnen M.F., Shih A.H., Zhou P., Kumar S., Soccio R.E., Kedersha N.L. (2000). The hPLIC proteins may provide a link between the ubiquitination machinery and the proteasome. Mol. Cell.

[116] Wang X., Chen C.-F., Baker P.R., Chen P., Kaiser P., Huang L. (2007). Mass spectrometric characterization of the affinity-purified human 26S proteasome complex. Biochemistry.

[117] Tai H.-C., Besche H., Goldberg A.L., Schuman E.M. (2010). Characterization of the Brain 26S Proteasome and its Interacting Proteins. Front. Mol. Neurosci..

[118] Clemens K.E., Brent R., Gyuris J., Munger K. (1995). Dimerization of the human papillomavirus E7 oncoprotein in vivo. Virology.

[119] Münger K., Grace M., Yee C., Jones D.L., Mavromatis K.O., Mukherjee R. (2002). The carboxyl-terminal zinc-binding domain of the human papillomavirus E7 protein can be functionally replaced by the homologous sequences of the E6 protein. Virus Res..

[120] Phelps W.C., Yee C.L., Münger K., Howley P.M. (1988). The human papillomavirus type 16 E7 gene encodes transactivation and transformation functions similar to those of adenovirus E1A. Cell.

[121] Phelps W.C., Munger K., Yee C.L., Barnes J.A., Howley P.M. (1992). Structure-Function Analysis of the Human Papillomavirus Type 16 E7 Oncoprotein. J. Virol..

[122] McLaughlin M., Münger K. (2010). The Human Papillomavirus E7 Oncoprotein. Virology.

[123] Roman A., Munger K. (2013). The papillomavirus E7 proteins. Virology.

[124] Smotkin D., Wettstein F.O. (1986). Transcription of human papillomavirus type 16 early genes in a cervical cancer and a cancer-derived cell line and identification of the E7 protein. Proc. Natl. Acad. Sci. USA.

[125] Morris E.J., Dyson N.J. (2001). Retinoblastoma protein partners. Adv. Cancer Res..

[126] Pintard L., Willems A., Peter M. (2004). Cullin-based ubiquitin ligases: Cul3-BTB complexes join the family. EMBO J..

[127] Zimmerman E.S., Schulman B.A., Zheng N. (2010). Structural assembly of cullin-RING ubiquitin ligase complexes. Curr. Opin. Struct. Biol..

[128] Xu J., Fang Y., Wang X., Wang F., Tian Q., Li Y., Xie X., Cheng X., Lu W. (2016). CUL2 overexpression driven by CUL2/E2F1/miR-424 regulatory loop promotes HPV16 E7 induced cervical carcinogenesis. Oncotarget.

[129] Westrich J.A., Warren C.J., Klausner M.J., Guo K., Liu C.-W., Santiago M.L., Pyeon D. (2018). Human Papillomavirus 16 E7 Stabilizes APOBEC3A Protein by Inhibiting Cullin 2-Dependent Protein Degradation. J. Virol..

[130] Vasudevan S., Starostina N.G., Kipreos E.T. (2007). The Caenorhabditis elegans cell-cycle regulator ZYG-11 defines a conserved family of CUL-2 complex components. EMBO Rep..

[131] Oh K., Kalinina A., Wang J., Nakayama K., Nakayama K.I., Bagchi S. (2004). The Papillomavirus E7 Oncoprotein Is Ubiquitinated by UbcH7 and Cullin 1- and Skp2-Containing E3 Ligase. J. Virol..

[132] Reinstein E., Scheffner M., Oren M., Ciechanover A., Schwartz A. (2000). Degradation of the E7 human papillomavirus oncoprotein by the ubiquitin-proteasome system: Targeting via ubiquitination of the N-terminal residue. Oncogene.

[133] Zhang B., Chen W., Roman A. (2006). The E7 proteins of low- and high-risk human papillomaviruses share the ability to target the pRB family member p130 for degradation. Proc. Natl. Acad. Sci. USA.

[134] Tedesco D., Lukas J., Reed S.I. (2002). The pRb-related protein p130 is regulated by phosphorylation-dependent proteolysis via the protein-ubiquitin ligase SCFSkp2. Genes Dev..

[135] Boyer S.N., Wazer D.E., Band V. (1996). E7 protein of human papilloma virus-16 induces degradation of retinoblastoma protein through the ubiquitin-proteasome pathway. Cancer Res..

[136] Jang J.-H. (2004). FIGC, a novel FGF-induced ubiquitin-protein ligase in gastric cancers. FEBS Lett..

[137] Ahmad K.F., Melnick A., Lax S., Bouchard D., Liu J., Kiang C.-L., Mayer S., Takahashi S., Licht J.D., Privé G.G. (2003). Mechanism of SMRT corepressor recruitment by the BCL6 BTB domain. Mol. Cell.

[138] Melnick A.M., Adelson K., Licht J.D. (2005). The theoretical basis of transcriptional therapy of cancer: Can it be put into practice?. J. Clin. Oncol..

[139] Kang M.-I., Kobayashi A., Wakabayashi N., Kim S.-G., Yamamoto M. (2004). Scaffolding of Keap1 to the actin cytoskeleton controls the function of Nrf2 as key regulator of cytoprotective phase 2 genes. Proc. Natl. Acad. Sci. USA.

[140] Bomont P., Cavalier L., Blondeau F., Ben Hamida C., Belal S., Tazir M., Demir E., Topaloglu H., Korinthenberg R., Tüysüz B. (2000). The gene encoding gigaxonin, a new member of the cytoskeletal BTB/kelch repeat family, is mutated in giant axonal neuropathy. Nat. Genet..

[141] Kobayashi A., Kang M.-I., Okawa H., Ohtsuji M., Zenke Y., Chiba T. (2004). Oxidative stress sensor Keap1 functions as an adaptor for Cul3-based E3 ligase to regulate proteasomal degradation of Nrf2. Mol. Cell. Biol..

[142] Pintard L., Willis J.H., Willems A., Johnson J.-L.F., Srayko M., Kurz T., Glaser S., Mains P.E., Tyers M., Bowerman B. (2003). The BTB protein MEL-26 is a substrate-specific adaptor of the CUL-3 ubiquitin-ligase. Nature.

[143] Geyer R., Wee S., Anderson S., Yates J., Wolf D.A. (2003). BTB/POZ domain proteins are putative substrate adaptors for cullin 3 ubiquitin ligases. Mol. Cell.

[144] Krek W. (2003). BTB proteins as henchmen of Cul3-based ubiquitin ligases. Nat. Cell Biol..

[145] Willems A.R., Schwab M., Tyers M. (2004). A hitchhiker’s guide to the cullin ubiquitin ligases: SCF and its kin. Biochim. Biophys. Acta.

[146] Chen Y., Yang Z., Meng M., Zhao Y., Dong N., Yan H., Liu L., Ding M., Peng H.B., Shao F. (2009). Cullin mediates degradation of RhoA through evolutionarily conserved BTB adaptors to control actin cytoskeleton structure and cell movement. Mol. Cell.

[147] Songock W.K., Kim S.-M., Bodily J.M. (2017). The human papillomavirus E7 oncoprotein as a regulator of transcription. Virus Res..

[148] Nakayama K., Nakayama N., Davidson B., Sheu J.J.-C., Jinawath N., Santillan A., Salani R., Bristow R.E., Morin P.J., Kurman R.J. (2006). A BTB/POZ protein, NAC-1, is related to tumor recurrence and is essential for tumor growth and survival. Proc. Natl. Acad. Sci. USA.

[149] Rahman M.T., Nakayama K., Rahman M., Katagiri H., Katagiri A., Ishibashi T., Ishikawa M., Iida K., Nakayama N., Otsuki Y. (2012). Fatty acid synthase expression associated with NAC1 is a potential therapeutic target in ovarian clear cell carcinomas. Br. J. Cancer.

[150] Sekine J., Nakatani E., Ohira K., Hideshima K., Kanno T., Nariai Y. (2015). Nucleus Accumbens-Associated Protein 1 Expression Has Potential as a Marker for Distinguishing Oral Epithelial Dysplasia and Squamous Cell Carcinoma. PLoS ONE.

[151] Basile J.R., Zacny V., Münger K. (2001). The Cytokines Tumor Necrosis Factor-α (TNF-α) and TNF-related Apoptosis-inducing Ligand Differentially Modulate Proliferation and Apoptotic Pathways in Human Keratinocytes Expressing the Human Papillomavirus-16 E7 Oncoprotein. J. Biol. Chem..

[152] ichiro Inoue J., Ishida T., Tsukamoto N., Kobayashi N., Naito A., Azuma S., Yamamoto T. (2000). Tumor necrosis factor receptor-associated factor (TRAF) family: Adapter proteins that mediate cytokine signaling. Exp. Cell Res..

[153] Cheng H., Zhan N., Ding D., Liu X., Zou X., Li K., Xia Y. (2015). HPV Type 16 Infection Switches Keratinocytes from Apoptotic to Proliferative Fate under TWEAK/Fn14 Interaction. J. Investig. Dermatol..

[154] Zhang J., Chen T., Yang X., Cheng H., Spath S.S., Clavijo P.E., Chen J., Silvin C., Issaeva N., Su X. (2018). Attenuated TRAF3 fosters activation of alternative NF-kB and reduced expression of antiviral interferon, TP53, and RB to promote HPV-positive head and neck cancers. Cancer Res..

[155] Santin A.D., Zhan F., Bignotti E., Siegel E.R., Cané S., Bellone S., Palmieri M., Anfossi S., Thomas M., Burnett A. (2005). Gene expression profiles of primary HPV16- and HPV18-infected early stage cervical cancers and normal cervical epithelium: Identification of novel candidate molecular markers for cervical cancer diagnosis and therapy. Virology.

[156] Lin C.H., Chang H.S., Yu W.C.Y. (2008). USP11 stabilizes HPV-16E7 and further modulates the E7 biological activity. J. Biol. Chem..

[157] Berezutskaya E., Bagchi S. (1997). The human papillomavirus E7 oncoprotein functionally interacts with the S4 subunit of the 26 S proteasome. J. Biol. Chem..

